# Personal

**Published:** 1905-07

**Authors:** 


### PERSONAL.

Dr. John B. Murphy, of Chicago, has been appointed professor of surgery at Rush medical college. He retains his place as chief surgeon of Mercy Hospital, but has resigned his position as professor of surgery at the Northwestern medical school.

---

Dr. J. D. Griffith, of Kansas City, Mo., recently spent a day in Buffalo. He was on his way home after attending the annual meeting of the American Orthopedic Society at Boston.

Dr. Griffith recently made a tour of the world and spent some time in our Philippine possessions. He also visited several of the Japanese military hospitals in different cities of the Island Empire. We hope to have the pleasure of publishing an account of Dr. Griffith's tour in a future number of the Journal.

---

Dr. C. W. Stranahan, of Erie, Pa., visited Buffalo for a day, about the middle of June. He will attend the meeting of the American Medical Association at Portland, and has secured tickets for himself and nephew over the Northwestern and Union Pacific lines.

---

Dr. F. Park Lewis, of Buffalo, was reëlected president of the board of managers of the New York State School for the Blind at Batavia, at the annual meeting held June 22, 1905.

It is announced that the board will take steps to introduce manual training into the curriculum of the school next year.

---

Dr. Samuel Wyllis Bandler, of New York, has been appointed adjunct professor of gynecology at the Post-Graduate medical school and hospital.

Dr. Robert T. Morris, of New York, has gone to the Hudson's Bay region for his summer vacation. He expects to return to his work about the middle of September.

Dr. John F. Winn, of Richmond, has been elected professor of clinical obstetrics at the University college of medicine in that city.

Dr. Francis E. Fronczak, of Buffalo, who attended M. Paderewski, when he was ill at Niagara Falls, and accompanied to Boston, remaining with him until he sailed for Europe, has received a letter from the famous pianist, saying he is at his home in Poland and rapidly improving.

Dr. Benjamin H. Grove, of Buffalo, has gone to the Pacific coast, and will attend the meeting of the A. M. A. at Portland. He will be absent a month.

